# High-Quality Perovskite CH_3_NH_3_PbI_3_ Thin Films for Solar Cells Prepared by Single-Source Thermal Evaporation Combined with Solvent Treatment

**DOI:** 10.3390/ma12081237

**Published:** 2019-04-15

**Authors:** Huanxin Peng, Zhenghua Su, Zhuanghao Zheng, Huabin Lan, Jingting Luo, Ping Fan, Guangxing Liang

**Affiliations:** Shenzhen Key Laboratory of Advanced Thin Films and Applications, College of Physics and Optoelectronic Engineering, Shenzhen University, Shenzhen 518060, China; P2385284535@163.com (H.P.); zhsu@szu.edu.cn (Z.S.); zhengzh@szu.edu.cn (Z.Z.); lanhb420@163.com (H.L.); luojt@szu.edu.cn (J.L.); fanping308@126.com (P.F.)

**Keywords:** evaporation, thin film, perovskite, solvent annealing

## Abstract

In this work, solvent annealing process for CH_3_NH_3_PbI_3_ thin film prepared by single source evaporation was reported. Characterized by the scanning electron microscope (SEM), X-ray diffractometer (XRD), energy dispersive spectroscope (EDS), ultraviolet-visible (UV) spectrophotometer, and the photoluminescence (PL) spectrometer, our method ensured higher quality film with crystallinity, composition, well-defined grain structure, and reproducibility. The optimized solar cell device based on the structure of ITO/PEDOT:PSS/CH_3_NH_3_PbI_3_/PCBM/Ag achieved better performance in power conversion efficiency from 2.64% to 9.92%, providing an effective method to optimize the quality of perovskite film for solar cell application.

## 1. Introduction

In recent years, owing to their low exciton binding energy, high carrier mobility, long carrier diffusion length, wide absorption spectrum, high optical absorption rate, and controllable composition, organic and inorganic hybrid metal halide perovskite materials have attracted much attention and quickly became one of the promising materials for solar cell application [1,2,3]. Their molecular formula could be expressed as ABX_3_ (A represents organic cation CH_3_NH_3_^+^, HC(NH_2_)_2_^+^, CH_3_CH_2_NH_3_^+^, or C(NH_2_)_3_^+^; B represents metal cation Pb^2+^ or Sn^2^; X represents halogen anion I^−^, Cl^−^, or Br^−^). It is unbelievable that the power conversion efficiency (PCE) of MAPbI_3_ solar cell has increased dramatically among all kinds of solar cells. Within a short time, from 2009 to 2016, the PCE of perovskite solar cells based on organic and inorganic hybrid metal halide perovskite (CH_3_NH_3_PbI_3_ (MAPbI_3_)) as absorber increases rapidly from 3.8% to 22.1%. Moreover, the latest research proved that the PCE of MAPbI_3_ solar cells could rise to 31% [4], which indicates that the development of MAPbI_3_ solar cells is not only limitless, but also has great potential for further exploration in solar cell application. As we all know, the thickness [5,6], size of crystal grains [7], elements stoichiometric ratio [8,9], uniformity, and coverage [10,11] of light absorption layer have a great influence on the performance of MAPbI_3_ solar cells, which directly affects the efficiency of perovskite solar cells. As for MAPbI_3_ absorber, the surface morphology of the thin film is much sensitive to the preparation processes. Recently, many fabrication methods have been applied for the preparation of the perovskite thin films, such as one-step solution, two-step solution, vacuum dual-source co-evaporation, vapor-assisted deposition and vacuum single-source thermal evaporation. Compared to solution methods, vacuum evaporation is not involved with solvent evaporation so that it could easily regulate and control preparation process and obtain the perovskite films with high homogeneity and uniformity. In this work, we adopted the vacuum single-source thermal evaporation, proposed by Fan et al. [12,13,14,15], to fabricate MAPbI_3_ films. Although the thin film was continuous with complete coverage, its crystal grains were small. Researchers found that perovskite films with micron-sized crystal grains is favorable for the carriers to separate and transport in the thin film [1,7,16,17,18,19,20] and solvent annealing is an effective method to promote the growth of the crystal grains [21,22]. For the sake of promoting the growth of grains, solvent annealing for MAPbI_3_ films with 1,4-Butyrolactone under atmospheric environment is an effective method [21,22]. In the air environment, the as-prepared perovskite films were annealed under solvent 1,4-Butyrolactone vapor and the solvent-annealed perovskite films with micron-sized grains were obtained. The effect of solvent-annealing process on the microstructure, composition, crystallinity, and optical properties of MAPbI_3_ thin films were investigated. Based on the solvent-annealed thin films, the perovskite solar cell achieved an optimized PCE from 2.64% to 9.92%.

## 2. Experimental Details

### 2.1. CH_3_NH_3_I and CH_3_NH_3_PbI_3_ Powder Preparation

CH_3_NH_3_I synthesis: We optimized the preparation process of CH_3_NH_3_I according to the references [23]. First, 28 mL methylamine (CH_3_NH_2_) (40 wt.% in water, Sigma Aldrich, St. Louis, MI, USA) reacted with 30 mL hydroiodic acid (HI) (57 wt.% in water, Sigma Aldrich) in the 250 mL round flask at 0 °C with constant magnetic stirring for 2 h. Then, we removed the reacted solution to the 500 mL beaker fixed on the water bath heated at 90 °C to attain white crystal power. Subsequently, the powder was purified using anhydrous ethanol and ether for three or four times. Last, the CH_3_NH_3_I powder was collected in the brown bottle and saved in the glove box before use after it was dried in the vacuum oven at 60 °C or 24 h.

Preparation of CH_3_NH_3_PbI_3_ powder: First, we weighed 9.22 g PbI_2_ powder and 3.14 g CH_3_NH_3_I powder, respectively, using the electronic balance and put them into the 20 mL γ-butyrolactone (≥99%, Sigma-Aldrich). Then, we removed the solution to the petri dish after ultrasonication for 30 min and heat it on the plate at 160 °C for 4 h. Finally, the CH_3_NH_3_PbI_3_ powder was collected in the brown box and stored in the glove box before use.

### 2.2. Film Preparation

First, the blank glass substrates were sequentially cleaned in the ultrasonic machine with deionized water, acetone and ethanol. Then, the glass substrates were dried by the nitrogen gun and stored in the drying oven for use. Later, we placed them into the substrate holder, close its baffle, and a certain amount of CH_3_NH_3_PbI_3_ powder was put into the evaporating boat. The deposition process of the CH_3_NH_3_PbI_3_ films is as follows. We turned on the heating power when the vacuum pressure of the chamber was down to 1.5 × 10^−3^ Pa. Then, we opened the baffle, smoothly increased the heating power to obtain the desired evaporating rate, and held it there for 15 min. The vertical distance between substrate holder and the evaporating boat was around 12 cm. After the perovskite films were done, they were thermally annealed at 150 °C for 20 min in a container of 500 mL under atmospheric environment. Meanwhile, 20 uL solvent γ-butyrolactone was evaporated in the closed container.

### 2.3. Device Fabrication

ITO (10 Ohm/sq, YINKOU OPVTECH, Yingkou, China) glass substrates were cleaned in the ultrasonic cleaning machine sequentially with the mixed solution of Decon 90, deionized water, and ethanol for 30 min, respectively. Then, the ITO substrates were dried by the nitrogen gun and stored in the drying oven for use. Prior to spin-coating the hole transport layer of PEDOT:PSS (CLEVIOS PVP AI4083, 70 nm), the ITO substrates were treated in the UV ozone cleaning system for 30 min. Then, the PEDOT:PSS layer was spin-coated on the substrates at 3000 rpm for 30 s. After that, the PEDOT:PSS-coated substrates were thermally annealed on a hot plate under the temperature of 180 °C for 20 min. The perovskite films were prepared by single-source vacuum thermal evaporation and the detailed information is in the Film Preparation. The electron transport layer (60 nm) of PCBM was spin-coated on the perovskite film at 3000 rpm for 30 s. The PCBM solution was prepared by dissolving 20 mg PCBM (>99%, Xi’an Polymer Light Technology Corp., Xi’an, China) powder in 1 mL chlorobenzene. Finally, a 150 nm thickness silver layer was deposited on the top of the device by thermal evaporation under the pressure of 1.5 × 10^−3^ Pa.

### 2.4. Characterization

The surface morphology of perovskite films was measured by high-resolution scanning electron microscope (SEM, SUPRA 55, Zeiss, Jena, Germany) with an accelerated voltage of 3 kV. The elemental analysis and the mapping patterns of the samples were measured by the energy dispersive X-ray microanalysis system (EDS) of Bruker QUANTAX 200 (Billerica, MA, USA) with an accelerated voltage of 15 kV. The X-ray diffraction (XRD) patterns of the samples were measured by the XRD system of Ultima IV (Rigaku, Tokgo, Japan) with CuKα radiation (0.15406 nm) operated at 40 kV and 40 mA. The optical absorption spectra of perovskite films were measured by the UV-vis spectrophotometer of Lambda 950, PerkinElmer (Waltham, MA, USA). The photoluminescence (PL) spectra of films were measured by the Raman spectrometer (inVia, Renishaw, Shanghai, China) with an excited wavelength of 532 nm (50 mW) and a spectral resolution of 1 cm^−1^. The device performance of the perovskite solar cell was tested using the Keithley 2400 (Keithley Instruments, Solon, OH, USA) under simulated AM 1.5G Solar Simulator (100 mW/cm^2^) under the ambient environment. The available active area of each solar cell is around 0.1 cm^2^. 

## 3. Results and Discussions 

**Figure 1:**
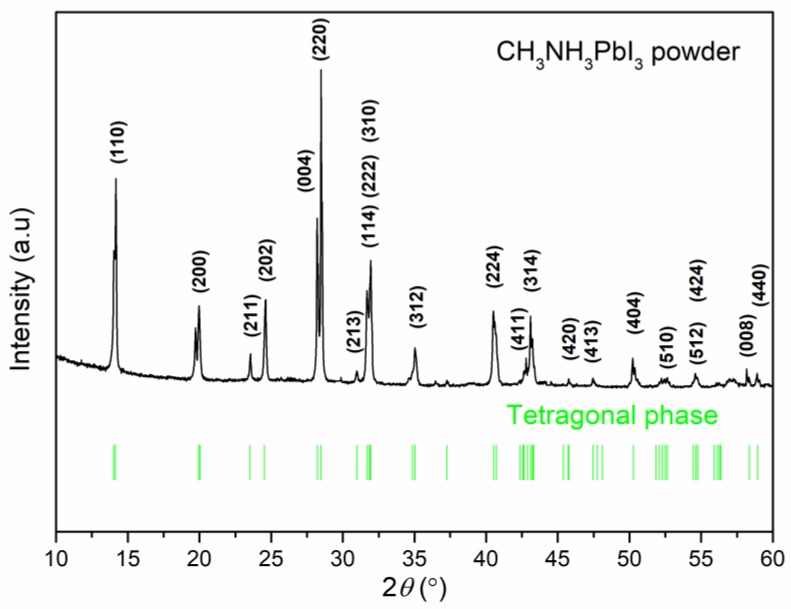
XRD pattern of the CH_3_NH_3_PbI_3_ powder and its corresponding data of tetragonal phase.

**Figure 2:**
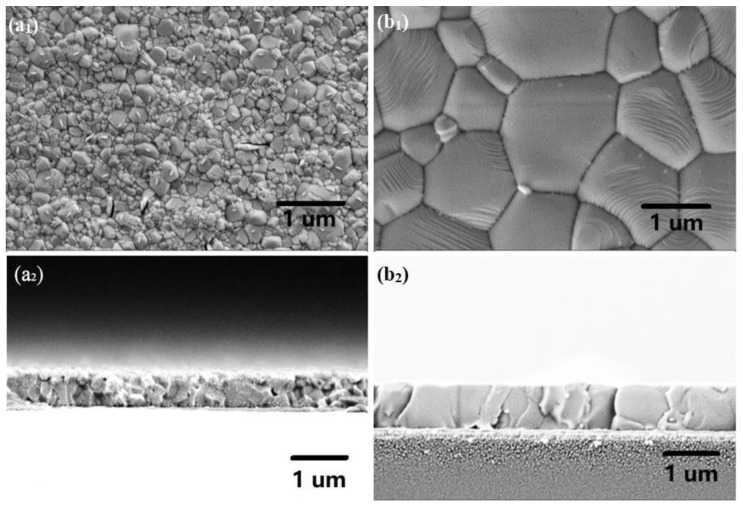
SEM images of as-prepared (**a_1_**: surface; **a_2_**: cross-section) and solvent-annealed (**b_1_**: surface; **b_2_**: cross-section) CH_3_NH_3_PbI_3_ films.

Figure 1 shows the XRD pattern of the CH_3_NH_3_PbI_3_ powder and its corresponding data of tetragonal phase. The XRD result of CH_3_NH_3_PbI_3_ powder was in well agreement with the CH_3_NH_3_PbI_3_ JCPDS data, indicating that CH_3_NH_3_PbI_3_ powder with tetragonal phase was obtained by our method in the Experimental details. Figure 2 shows the surface and cross-section morphology of as-prepared and solvent-annealed CH_3_NH_3_PbI_3_ films. All the CH_3_NH_3_PbI_3_ thin films exhibited full surface coverage. However, the average grain size was mostly below 500 nm for as-prepared thin films while the solvent-annealed was up to 1 μm. The perovskite films were thermally annealed in the solvent vapor atmosphere in a sealed container. With the suitable usage of solvent evaporated, the solvent vapor could go into the grain boundaries and partially dissolve the perovskite film. Meanwhile, the perovskite films could recrystallize as the solvent was re-evaporated under the high annealing temperature. In addition, the solvent-annealed film showed a smoother surface with bigger crystal grains compared to as-prepared, which could lead to fewer crystal boundaries and accelerate the charge extraction process. From the cross-section images of CH_3_NH_3_PbI_3_ films, it can be found that the larger grains were evenly distributed with the size over film thickness which was beneficial to significantly decrease charge recombination loss between boundaries.

**Figure 3:**
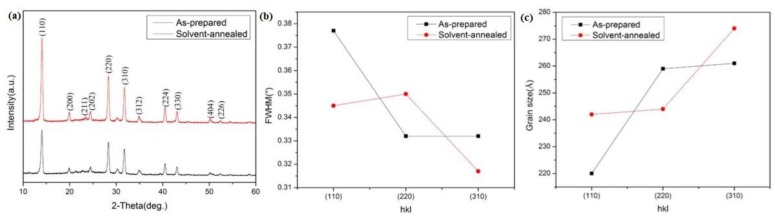
XRD patterns (**a**) and FWHM (**b**) and average crystalline size (**c**) of as-prepared and solvent-annealed films.

Figure 3 shows the X-ray diffraction (XRD) pattern and its corresponding FWHM and average crystal size of as-prepared and solvent-annealed CH_3_NH_3_PbI_3_ films. All the samples exhibited intense diffraction peaks at 14.02°, 28.32°, and 31.76°, respectively assigned to the (110), (220), and (310) planes of tetragonal perovskite structure [24], which indicated that the desired perovskite film was formed. Obviously, the main peak intensity of the solvent-annealed CH_3_NH_3_PbI_3_ film was stronger and sharper than that the as-prepared, which was consistent with the trend of FWHM and shown in Figure 3b. The average crystalline size of (110), (220), and (310) planes in the as-deposited and solvent-annealed film was estimated by resolving the characteristic peaks using Scherrer’s formula

(1) D = Kγ/(B×cosθ)

where *K* is Scherrer constant, *D* is the average grain size, *B* is the half-width of the measured diffraction peak, *θ* is the diffraction angle, and *γ* is the X-ray wavelength (0.154056 nm).

It can be seen from the Figure 3c that the average crystal size of solvent-annealed perovskite film became bigger. The more intense characteristic peaks and larger average crystal size both attributed to the improved crystallinity and/or composition homogeneity with fewer low-dimensional defects and less scattering between grain boundaries in the solvent-annealed perovskite film.

**Figure 4:**
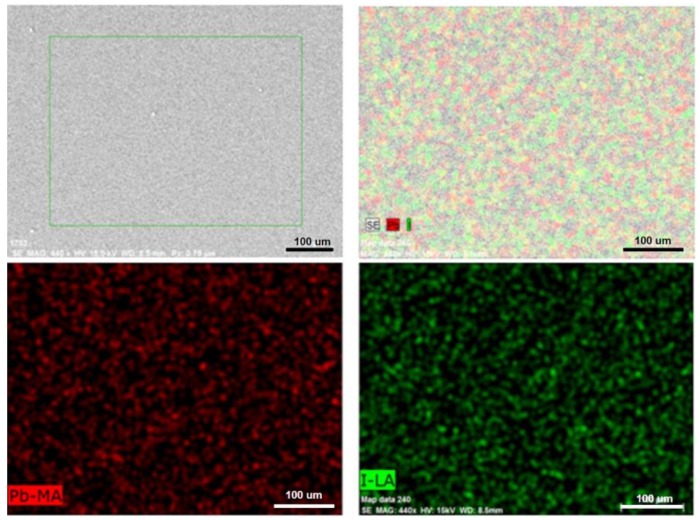
The elemental mapping of Pb and I in the solvent-annealed CH_3_NH_3_PbI_3_ film.

The elemental ratio of Pb and I of the as-prepared and solvent-annealed CH_3_NH_3_PbI_3_ films measured by EDS is shown in Table 1. The Pb/I of as-prepared and solvent-annealed CH_3_NH_3_PbI_3_ films, respectively, were 1/3.04 and 1/3.03, which was in well agreement with the theoretical stoichiometry of 1/3. In order to further examine the compositional distribution throughout the solvent-annealed films, Figure 4 shows the elemental mapping of Pb and I with uniformly distributed, indicating that the solvent annealing is a reliable method to fabricate the desired perovskite film.

**Figure 5:**
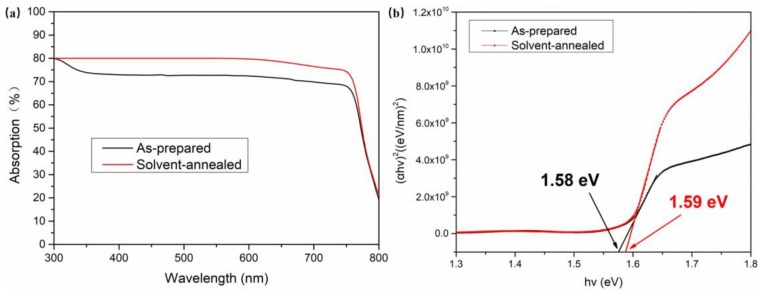
(**a**) Optical absorption spectra and (**b**) the corresponding Tauc plots of the as-prepared and solvent-annealed CH_3_NH_3_PbI_3_ films.

**Figure 6:**
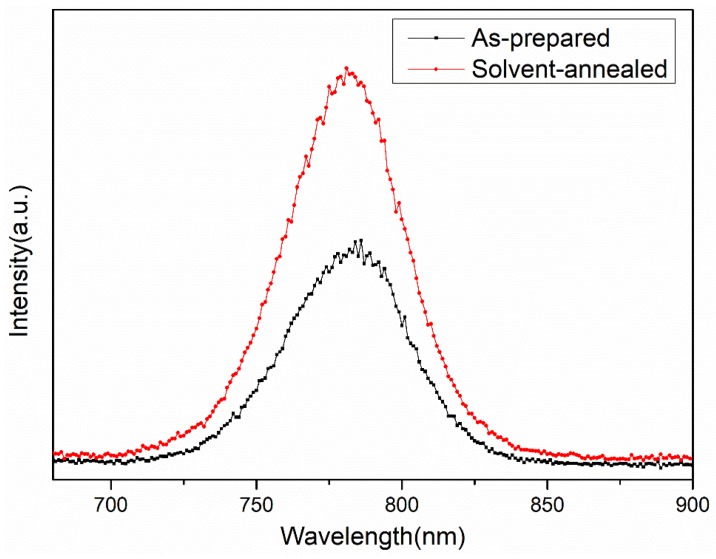
Photoluminescence (PL) spectra of CH_3_NH_3_PbI_3_ films with and without solvent annealing.

Figure 5 shows the optical absorption spectra and the corresponding Tauc plots of the as-prepared and solvent-annealed MAPbI_3_ films. The optical absorption of the as-prepared perovskite film was much lower than that of the solvent-annealed in the wavelength range from 300 nm to 750 nm because of more defect states caused by the smaller grains, as shown in the SEM images. Additionally, all the samples showed a sharp absorption edge in the absorption spectra at about 780 nm. In addition, the optical band-gap could be calculated according to the following equation

(2) $${\left(\alpha hv\right)}^{2}=A\left(hv-E_{g}\right)$$

where A is a constant that is uncorrelated with the value of the optical bandgap, hv is the photon energy, α is the optical absorption coefficient obtained from the measured absorption spectrum, and $E_{g}$ is the bandgap of the light absorbing material. The calculated optical band gap of the as-prepared and solvent-annealed CH_3_NH_3_PbI_3_ films were 1.58 eV and 1.59 eV, respectively, which was consistent with the reported for MAPbI_3_ [25]. Figure 6 shows the Photoluminescence (PL) spectra of CH_3_NH_3_PbI_3_ films with and without solvent annealing. It can be observed that the PL peaks of CH_3_NH_3_PbI_3_ films with and without solvent annealing were respectively located at 781 and 785 nm, which agreed with the above calculated bandgap result. In addition, the intensity of the PL peak of CH_3_NH_3_PbI_3_ film with solvent annealing was incredibly stronger than that of as-prepared perovskite film, suggesting that the number of defects and trap states of CH_3_NH_3_PbI_3_ film reduced after solvent annealing.

**Figure 7:**
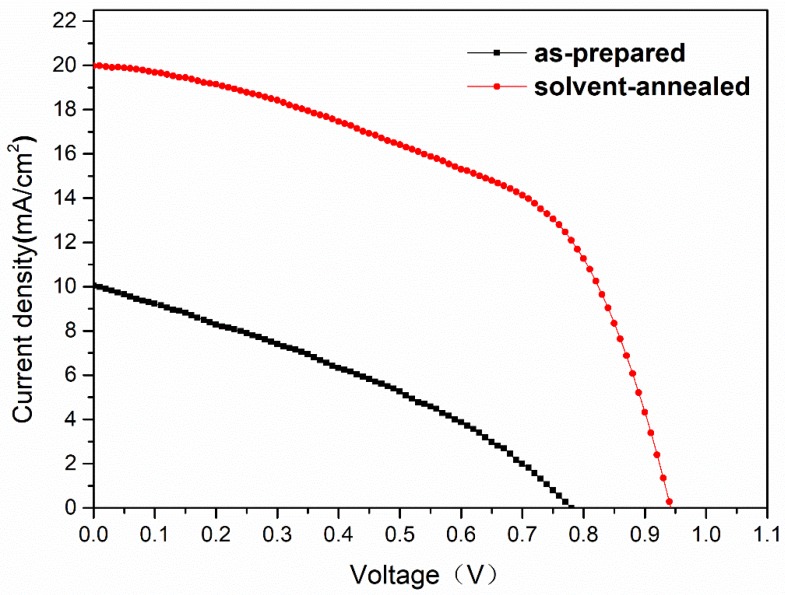
The J-V curves of as-prepared and solvent-annealed MAPbI_3_ solar cells.

**Figure 8:**
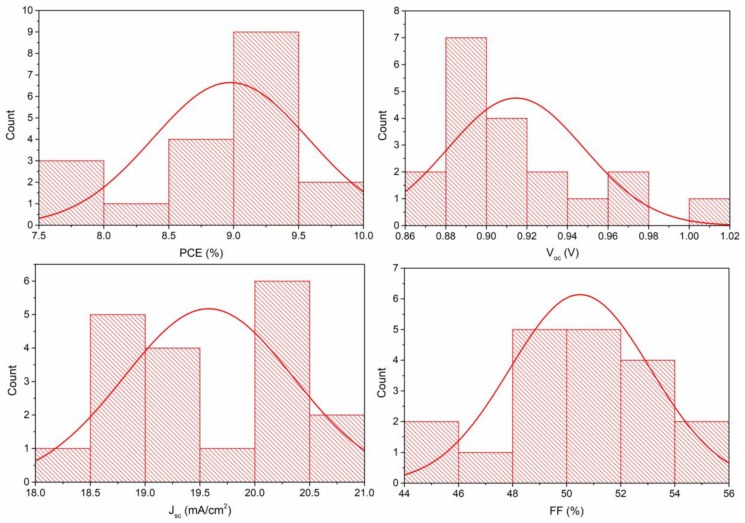
The histograms of the PCE, V_oc_, J_sc_, and FF distribution based on 19 perovskite solar cells.

Figure 7 shows the J-V curves of CH_3_NH_3_PbI_3_ solar cells. The related open-circuit voltage (V_oc_), short-circuit current density (J_sc_), fill factor (FF), and PCE are summarized in Table 2. It could be inferred from the figure that the performance of solvent-annealed perovskite solar cell with V_oc_ of 0.94 V, J_sc_ of 19.97 mA/cm^2^, FF of 53%, and PCE of 9.92% was much better than that of as-prepared one with V_oc_ of 0.77 V, J_sc_ of 10.04 mA/cm^2^, FF of 34%, and PCE of 2.64%. Here, the reason why the fill factors (FF) of the devices were low may be the presence of the underestimated defects in the interface, especially between the absorption layer and the hole transport layer, leading to carrier recombination loss during electron transporting. Figure 8 shows the histograms of the PCE, V_oc_, J_sc_ and FF distribution based on 19 perovskite solar cells. The devices based on the solvent-annealed films showed high reproducibility with average PCE of 8.98 ± 0.60%. The improved device performance and high reproducibility were contributed to the increased optical absorption, micron-sized crystal grain and fewer defects of CH_3_NH_3_PbI_3_ film after solvent-annealing treatment, which indicated that the solvent annealing was a promising method to significantly improve the quality of perovskite film and enhanced the efficiency of CH_3_NH_3_PbI_3_ solar cells. 

## 4. Conclusions

In this paper, after the solvent annealing process, larger grains, higher crystallinity, well-defined grain structure, desired element stoichiometric ratio, and excellent optical properties of CH_3_NH_3_PbI_3_ films prepared by single source evaporation can be obtained. Solar cell devices based on the perovskite films with solvent-annealing treatment achieved a much higher power conversion efficiency of 9.92% than that of 2.64% of as-prepared, suggesting that single-source vacuum thermal evaporation assembled with the solvent annealing was a potential method to prepare the high-quality perovskite films for photovoltaic application.

## Figures and Tables

**Table 1:** The elemental ratio of Pb and I in as-prepared and solvent-annealed CH_3_NH_3_PbI_3_ films measured by energy dispersive X-ray microanalysis system (EDS).

Sample	Pb (at.%)	I (at.%)	Pb/I
As-deposited films	24.75	75.25	1/3.04
Annealed films	24.76	75.24	1/3.03

**Table 2:** The performance of the as-prepared and solvent-annealed MAPbI_3_ solar cells.

Sample	J_sc_ (mA/cm^2^)	V_oc_ (V)	FF (%)	PCE (%)
As-prepared	10.04	0.77	34	2.64
Solvent-annealed	19.97	0.94	53	9.92
